# New insights into the role of immunity and inflammation in diabetic kidney disease in the omics era

**DOI:** 10.3389/fimmu.2024.1342837

**Published:** 2024-02-29

**Authors:** Xinrong Hu, Sixiu Chen, Siyang Ye, Wei Chen, Yi Zhou

**Affiliations:** ^1^ Department of Nephrology, The First Affiliated Hospital, Sun Yat-sen University, Guangzhou, China; ^2^ Key Laboratory of Nephrology, National Health Commission and Guangdong Province, Guangzhou, China

**Keywords:** diabetic kidney disease, inflammation, genomics, epigenomics, transcriptomics, proteomics, metabolomics

## Abstract

Diabetic kidney disease (DKD) is becoming the leading cause of chronic kidney disease, especially in the industrialized world. Despite mounting evidence has demonstrated that immunity and inflammation are highly involved in the pathogenesis and progression of DKD, the underlying mechanisms remain incompletely understood. Substantial molecules, signaling pathways, and cell types participate in DKD inflammation, by integrating into a complex regulatory network. Most of the studies have focused on individual components, without presenting their importance in the global or system-based processes, which largely hinders clinical translation. Besides, conventional technologies failed to monitor the different behaviors of resident renal cells and immune cells, making it difficult to understand their contributions to inflammation in DKD. Recently, the advancement of omics technologies including genomics, epigenomics, transcriptomics, proteomics, and metabolomics has revolutionized biomedical research, which allows an unbiased global analysis of changes in DNA, RNA, proteins, and metabolites in disease settings, even at single-cell and spatial resolutions. They help us to identify critical regulators of inflammation processes and provide an overview of cell heterogeneity in DKD. This review aims to summarize the application of multiple omics in the field of DKD and emphasize the latest evidence on the interplay of inflammation and DKD revealed by these technologies, which will provide new insights into the role of inflammation in the pathogenesis of DKD and lead to the development of novel therapeutic approaches and diagnostic biomarkers.

## Introduction

Diabetic kidney disease (DKD) encompasses the spectrum of people with diabetes mellitus (DM) (both type 1 and type 2 [T1DM and T2DM]) who manifest specific pathologic structural and functional changes in kidneys that result from DM (1). Although only 30% to 40% of DM patients develop DKD, it has become the leading cause of chronic kidney disease (CKD) and accounts for up to 50% of the end-stage renal disease (ESRD) population in Western countries (2). DKD is traditionally regarded as a microvascular complication induced by hyperglycemia and hemodynamic changes, mainly in glomeruli (3). Until recent decades, the important role of chronic inflammation and immune cells has been recognized in the pathogenesis and progression of DKD (4). Elevated pro-inflammatory cytokines were detected in serum, urine, and renal tissue from DM patients (5). Infiltrating immune cells are commonly found in renal biopsy samples at all stages of DKD, both in the glomeruli and interstitium (6). Macrophage occupies the majority of these cells, which secretes various cytokines to promote inflammation and fibrosis (7). T lymphocytes, recruited to the diabetic kidney accompanying macrophages, further exacerbate renal inflammation and dysfunction (8). Other cells, including B lymphocytes (9), dendritic cells (10), natural killer cells (11), mast cells (12), etc., are less explored and little is known about their roles in DKD.

However, it remains a challenge to decipher the complex interplay between immune cells and renal cells, as well as the mechanism underlying the unresolved inflammation that involves multiple signaling pathways and cytokines. The emergence of omics technology has brought unprecedented resolution, breadth, and depth to the inspection of biological systems, empowering research on immune- or inflammation-related pathogenic mechanisms in DKD (13, 14). Moreover, the integration of multi-omics (e.g., genome, epigenome, transcriptome, proteome, and metabolome), which often have complementary and synergistic effects, holds the key to acquiring a new and incomparable level of understanding of the nephropathy, building predictive models of DKD, and finding novel therapeutic targets taking advantage of the renal-immune interplay.

## The pathobiology of inflammation is incompletely understood in DKD

Mounting evidence has indicated the involvement of inflammation in the pathogenesis and progression of DKD (15). Preclinical studies have demonstrated that multiple inflammatory signaling pathways are activated in renal cells as responses to hyperglycemic insults, such as Toll-like receptor (TLR), nucleotide-binding oligomerization domain (NOD)-like receptor (NLR), nuclear factor-kappa B (NF-κB), Janus kinase/signal transducer and activator of transcription (JAK-STAT) signaling pathway, etc (16). As a result, these cells robustly produce pro-inflammatory molecules including cytokines (interleukin-6 [IL-6], tumor necrosis factor [TNF-α], IL-17A, IL-1β), chemokines (C-C chemokine receptor 2 [CCR2], C-C motif chemokine ligand 2 [CCL2], C-X-C motif chemokine ligand 10 [CXCL10], CXCL12), and adhesion molecules (αVβ3 integrin, intercellular adhesion molecule 1 [ICAM-1], galectin-3), which recruit several immune cells into kidneys and amplify inflammation, ultimately damaging renal function (17–20). Consistently, studies in diabetic patients also observed histological evidence of kidney inflammation and elevated levels of pro-inflammatory mediators (21, 22). Moreover, agents targeting inflammatory responses and immune cells have shown beneficial effects on diabetic animal models (23), which prompts their application in DM patients to improve renal outcomes.

However, many of the promising anti-inflammatory drugs have failed to effectively treat DKD in clinical trials, reflecting an inadequate understanding of how inflammation contributes to the development of the disease (24). Current knowledge regarding the pathobiology of inflammation cannot explain the heterogeneity in kidney manifestations among patients (25). Moreover, since inflammation might play divergent roles in different phenotypes and at various stages of the disease, it remains a challenge to identify effective anti-inflammatory targets for most DKD patients. Besides, the differences in immune cell phenotypes of mice and humans also partly contribute to the discrepancies between experimental models and clinical trials, highlighting the need to incorporate human samples in preclinical studies (26). The promise of the multiple omics approach to decipher the underlying molecular mechanisms of disease phenotypes has been well-described (27, 28). Thus, research integrating omics layers (genomics, epigenomics, transcriptomics, proteomics, metabolomics) in cohorts of DKD patients will enable us to further understand the complexity of this disease, as well as provide insights into novel therapeutic strategies.

## Genomics

The importance of genetic risk factors in DKD has been established by pedigree studies showing familial aggregation of nephropathies in DM patients (29). Diabetic individuals with DKD parents or siblings have an increased risk of nephropathy (30). Besides, the prevalence of DKD dramatically varies among different ethnic groups (31). This evidence prompts the search for specific genetic variants that confer susceptibility or progression for DKD. Several consortia dedicated to addressing the genetic basis of DKD have been initiated across the world, including Family Investigation of Nephropathy and Diabetes (FIND), GEnetics of Nephropathy — an International Effort (GENIE), and Genetics of Kidneys in Diabetes Study (GoKIND), along with many individual research groups (27). Benefiting from the advent of genome-wide association studies (GWAS), the identification of plausible and reproducible genetic associations can be based on an unbiased systematic screen, enhancing the chances of discovering genuine DKD risk variants.

The largest GWAS study of the risk loci for subjects with T1DM was launched by GENIE, in which they found two single nucleotide polymorphisms (SNPs) were associated with ESRD: one in *AFF3* gene (*P* = 1.2 × 10^−8^) and the other between the genes *RGMA* and *MCTP2* (*P* = 2.0 × 10^−9^) (32). The mRNA expression of *AFF3* is known to be relatively restricted to lymphocytes and brain tissues, encoding the AFF3 protein that facilitates the class switch recombination in B lymphocytes (33). *AFF3* was also recognized as a candidate gene for rheumatoid arthritis, suggesting its modulatory effects on the immune system and inflammation that may contribute to the development of DKD (34). While polymorphisms of RGMA were associated with different expression levels of interferon-γ (IFN-γ), TNF, and IL-21 receptor (IL-21R) in other inflammatory diseases (35). Additionally, the GWAS study involving the hugest sample size of T2DM patients discovered *GABRR1* (*P* = 4.5 × 10^−8^) as a novel locus associated with microalbuminuria in European individuals (36). The major allele in DKD patients is associated with decreased expression of *GABRR1* which could exert anti-inflammatory actions in post-ischemic brains (37). Winkler et al. identified 29 gene loci potentially responsible for reduced estimated glomerular filtration rate (eGFR) in diabetic patients, 27 of which were newly discovered (38). Among them, *UMOD* (*P* < 5 × 10^−8^), encoding uromodulin that was synthesized and secreted by renal tubular epithelial cells, was reported to have immunomodulatory effects. A previous study revealed that uromodulin triggered the activation of TLR4 signaling in renal dendritic cells (38). Uromodulin also served as an NLRP3 agonist in innate immune cells and promoted the secretion of IL-1β (39). However, the relationship between uromodulin and eGFR was not confined to DKD. Higher UMOD levels were also associated with smaller eGFR declines in CKD patients (40). Over the past decades, substantial studies have constantly revealed new inflammatory genes associated with DKD. A systematic review and meta-analysis based on 103 GWAS studies summarized multiple well-recognized inflammatory genes as risk variants for DKD, including *CCL2*, *CCR5*, *IL6*, *IL8*, *IL1A*, *IL1B*, *TNF*, *TNFRSF19*, etc (41).

Despite many successes with GWAS, the majority of identified variants map to noncoding regions with unknown effects (42). Moreover, these studies do not adequately interpret the disease-causing genes and mechanisms, only highlighting the relevance of inflammation in DKD. To address these challenges, transcriptome-wide association studies (TWAS) have recently emerged as a promising approach for prioritizing causal genes at GWAS loci (43). TWAS integrates GWAS with expression quantitative trait loci (eQTL) which informs the association between genetic variant and gene expression (44). Therefore, TWAS is capable of detecting functional gene expression regulated by DKD-associated variants, thus providing insight into the mechanisms of the diseases. Successful use of TWAS identified that the eGFR-associated SNP rs626277 regulated the expression of *DACH1*, which was validated in human and mouse kidney single-cell open chromatin data (scATAC-seq) (45). The functional study illustrated that loss of *DACH1* was associated with the pro-inflammatory phenotype of tubular cells, which released cytokines including CCL2 and macrophage colony stimulating fator-1 (CSF-1), leading to macrophage infiltration and severe fibrosis in diabetic kidneys. Apart from eQTL, methylation quantitative trait locus (mQTL), which conveys the relationships between DNA sequence variation and DNA methylation, can also be used to prioritize GWAS loci (46, 47). A complete study that incorporated GWAS, eQTL, and mQTL illustrated that the eGFR-associated GWAS SNP rs3757387 mediated methylation changes of cg0486179, which affects the expression of *IRF5*, a central regulator of the inflammatory response (48, 49). Indeed, 31 high-credibility protein-coding genes associated with kidney function in DKD were identified through this integration analysis, which showed significant enrichment for immune response in gene ontology analysis. These studies not only suggest the causal role of inflammation in DKD but also implicate the value of genomics as a practical tool for discovering risk variants (**Table 1**). Furthermore, they advance our understanding of the associations between genotypes with phenotypes, which helps to better understand DKD etiology and identify potential drug targets.

**Table 1:** Immune or inflammation-related genes in DKD identified by genomics.

Gene	SNP	Function	References
***AFF3***	rs7583877	Lymphoid development and oncogenesis.	Sandholm N et al. (2012) (32)
***GABRR1***	rs9942471	Anti-inflammation	van Zuydam et al. (2018) (36)
***UMOD***	rs77924615	Pro-inflammation	Winkler et al. (2022) (38)
***CCL2***	rs3917887	Chemokine	Tziastoudi et al. (2017) (41)
***CCR5***	rs1799987	Chemokine receptor	
***IL6***	rs1800796	Pro-inflammatory cytokine	
***IL8***	rs4073		
***IL1A***	rs1800587		
***IL1B***	rs16944		
***TNF***	rs1800629		
***TNFRSF19***	rs9510795		
***DACH1***	rs626277	Pro-inflammatory cytokine transcriptional repressor	Doke et al. (2021) (45)
***IRF5***	rs3757387	IFN-responsive transcription factor	Sheng et al. (2020) (48)

## Epigenetics

Though therapeutics targeting hyperglycemia have greatly improved renal outcomes of DM patients, the correlation between metabolic control and kidney disease is relatively poor (50, 51). Patients who once had poor glycemic control exhibited an increased risk for nephropathy even after decades of adequate glycemic control. This phenomenon is termed metabolic memory, which is primarily mediated by the epigenetic reprogramming of DKD-related genes (52). Thereby, hyperglycemia results in long-lasting transcriptional regulation of genes, many of which are involved in the activation of immune and inflammatory responses (53).

A previous GWAS study has identified a strong association between engulfment and cell motility 1 gene (*EMOL1*) and susceptibility to DKD (54). Further methylome analysis revealed differentially methylated CpG sites in the enhancer regions of *EMOL1*, which may be responsible for the increase in expression of *EMOL1* in patients (48). In more recent research, the diabetic mouse with different expression of *EMOL1* ranging from ∼30% to ∼200% normal was utilized (55). They showed that the severity of nephropathy was paralleled with the expression of *EMOL1*. Besides, reduced *EMOL1* expression blocked the development of diabetic nephropathy. EMOL1 is critical for innate immunity since it is indispensable for the clearance of apoptotic cells and pathogens as well as for the control of inflammatory responses (56). It is reported that loss of *EMOL1* mitigated neutrophil recruitment and inflammatory arthritis, indicating that the protective role of *Emol1* knock-down in DKD was partly due to its anti-inflammatory effects (57). Park et al. generated whole-genome DNA methylation maps for kidney samples from healthy and DKD patients (58). Gene set enrichment analysis of RNA sequencing data revealed that genes with differential methylation in DKD samples were enriched in TNF signaling. Lower methylation levels of *TNF* promoter led to higher expression of *TNF* in DKD, highlighting the epigenetic regulation of inflammation in the disease.

Given the essential role of immune cells regulating in DKD inflammation, several research focused on the epigenetic reprogramming of these cells. DNA methyltransferase 1 (DNMT1), a key enzyme for DNA methylation, was found to increase along with the inflammatory activity of peripheral blood mononuclear cells (PBMC) in DKD patients (59). Methylome analysis identified the differentially methylated cytosines in mammalian target of rapamycin (mTOR) gene promoters in PBMC, suggesting the involvement of epigenetic regulation of the pathogenic activation of the mTOR pathway in immune cells. Moreover, the acetylation of histone H3 at TNF-α was elevated in macrophages cultured in high-glucose (HG) conditions and PBMC from DM patients (60). The epigenetic modifications by HG could persist for a long term and induce the hyperglycemic memory immune cells, which is called trained immunity (61). Edgar et al. discovered that HG promoted pro-inflammatory gene expression in macrophages by increasing histone 3 Lys4 trimethylation and histone 3 Lys27 acetylation (62). These epigenetic features were retained in macrophages even when cultured in a normal glucose medium, denoting hyperglycemia-induced trained immunity. Therefore, intensive glucose control is not sufficient to suppress the pro-inflammatory phenotype of immune cells. This finding promotes our mechanistic understanding of the non-responders to glucose-lowering agents, also highlights that alternative therapies targeting epigenetic modifications are critical for this population. However, the involvement of trained immunity in diabetic nephropathy needs further validation.

Integration of epigenomics with genomics and transcriptomics facilitates the functional annotation of the identified differentially methylated or acetylated sites, however, few studies have accomplished it. Although massive epigenetic modifications have been unearthed by the high-throughput method in cohorts of DKD patients, their mechanistic regulation of the inflammation is inadequately researched. Suffering from this, the development of anti-inflammatory therapeutics targeting epigenetic mechanisms was largely limited in DKD.

## Transcriptomics

Numerous single-cell studies have been done on DKD, however, since single-cell transcriptome assays require a sufficient number of cells for in-depth analysis, most studies have focused on the alterations of renal resident cells, such as proximal tubules (PT), mesangial cells, and endothelial cells (63). Nonetheless, single-cell studies have explored the landscape of immune cells in DKD tissues. Multiple single-cell RNA sequencing (scRNA-seq) studies have demonstrated the elevated proportion of immune cells during the progression of DKD (7, 64–69). Chen et al. (65) using a combination of scRNA-seq and spatial transcriptomics found that immune cells were predominantly enriched in areas of renal fibrosis. Furthermore, a study that employed single-nucleus RNA sequencing (snRNA-seq) in uninephrectomized (UNx) *db/db* mouse models of early-stage (UNx only) and advanced DKD (UNx-Renin), detected the gene expression changes associated with metabolism were more prominent in early-stage DKD, whereas immune responses were more conspicuous in advanced DKD (66). These findings collectively point to the significant role of the immune response in the progression of DKD.

The composition of immune cells in DKD varies among datasets, which could be attributed to different dissociation methods and types of tissues used. In datasets from DKD mouse models, macrophages were identified as the predominant immune cells. Further analysis demonstrated the number of macrophages expressing M1 phenotypic markers increases as DKD progresses (65, 66, 70). The limited number of immune cells in the whole kidney has prevented in-depth analysis of immune cell functions and mechanisms by single-cell transcriptomics, thus Fu et al. (7) enriched CD45^+^ immune cells in mouse kidney tissues of the early and advanced stages of DKD. This study further focused on macrophages and revealed the dynamic changes of macrophage subclusters during the progression of DKD (**Table 2**). During the early stages of DKD, the increase of infiltrating macrophages, high-interferon (IFN) signature macrophages, and macrophage subsets with high expression of Mannose Receptor C-Type 1 (MRC1) or Triggering Receptor Expressed on Myeloid Cells 2 (TREM2), suggesting that both pro- and anti-inflammatory pathways are concomitantly regulated in macrophages during the early stages of DKD. In addition, a subset of macrophage called “M14” was identified, showing increased proportion in 7-month-old OVE26 kidneys. The expression profile of M14 bears similarities to that of Mrc1^hi^ macrophages but is characterized by higher expression levels of classic M2 macrophage markers. Furthermore, marker genes associated with M14, namely *FCN1*, *CD209*, and *FOLR2*, were found to be upregulated in human DKD through deconvolution of bulk RNA-seq dataset. This discovery is particularly intriguing, and the roles of this subset of macrophages need to be further clarified. Meanwhile, a study of scRNA-seq in *db/db* mice treated with angiotensin receptor blockers (ARBs) or sodium-glucose cotransporter 2 inhibitors (SGLT2i) demonstrated that treatment with ARBs or SGLT2i restored the proportion of macrophages in kidney tissues of DKD mice, especially ARBs (67). Overall, the dynamics of macrophages in the progression of DKD were unveiled, shedding light on the intricate regulation of pro- and anti-inflammatory pathways in macrophages during the disease.

**Table 2:** Changes in macrophage subclusters and related gene markers identified by Fu et al. (7) in the early- and late-stage of DKD.

Time point	Cell type	Proportion fold change to WT	Markers (top 10)
Early-stage DKD	IFN^hi^ Mac	1.4	*Cxcl9, Cxcl10, Isg15, Ccl12, Gbp2b, Iigp1, Gbp2, Serpina3g, Ifi47, Ifit2*
	Infiltrating Mac	1.23	*Retnla, S100a6, S100a4, Plac8, Fn1, Msrb1, Chil3, Itgal, Pglyrp1, Ear2*
	Trem2^hi^ Mac	1.21	*Fxyd2, Car2, Hpgd, Cyb5a, Lyz2, Cd14, Capg, Plxdc2, Ccl4, Pkm*
	Mrc1^hi^ Mac	1.16	*Pf4, Ccl12, Ms4a7, Apoe, Ccl8, Selenop, Mrc1, Vcam1, Slc40a1, Ms4a6b*
	Resident Mac	0.92	*Mmp13, Tgfbr1, Mgl2, Lifr, Lilra5, Maf, Selenop, Ctsh, P2ry6, Cx3cr1*
	Inflammatory Mac	0.88	*Ccl4, Ccl3, Il1b, Cxcl2, Hspa1a, Bcl2a1b, Marcksl1, Ccl12, Cd72, Bcl2a1a*
	Proliferating Mac	0.76	*Stmn1, Mcm6, Tubb5, Mcm3, Gmnn, Hmgb2, Hells, Dut, Tuba1b, Ranbp1*
Late-stage DKD	M14	2.85	*Retnla, Fcna, F13a1, Ccl8, Cd209f, Folr2, Lyve1, Hal, C4b, Ccl24*
	Infiltrating Mac (Ly6c^lo^)	1.45	*Ace, Itgal, Pglyrp1, Ear2, Msrb1, Samsn1, Gngt2, Cebpb, Ear1, Ceacam1*
	Trem2^hi^ Mac	1.4	*Fxyd2, Car2, Hpgd, Lyz2, Plxdc2, Gas7, Cyb5a, Cd14, Cst3, Pmepa1*
	Mrc1^hi^ Mac	1.24	*Vcam1, Mrc1, Selenop, Stab1, Pf4, Maf, Wwp1, Ms4a7, Apoe, Dab2*
	Infiltrating Mac (Ly6c^hi^)	1.08	*Fn1, Chil3, Ahnak, F13a1, S100a6, Ly6c2, Plac8, S100a4, Vim, Ifitm6*
	Inflammatory Mac	1.05	*Ccl4, Lpl, Il1b, Mmp12, Cd83, Cd72, Stap1, Wfdc17, Tnfaip2, Slc15a3*
	IFN^hi^ Mac	0.69	*Cxcl9, Isg15, Ifit2, Iigp1, Ccl12, Serpina3g, Gbp2, Oasl2, Slfn5, Ifi202b*
	Resident Mac	0.65	*Slamf9, C1qa, Tgfbr1, C1qb, Aif1, Mgl2, C1qc, Ifi27l2a, Gpr65, Tmem176b*

The study of immune cells other than macrophages in DKD has been relatively limited, possibly due to the small number of these cells detected in scRNA-seq/snRNA-seq. Wilson et al. (68, 69) conducted snRNA-seq based on cryopreserved human diabetic kidney samples, and found that diabetics exhibited an increased number of leukocytes, including T lymphocytes (49%), B lymphocytes (21%), monocytes (23%), and plasma cells (7%). Additionally, the presence of IgG^+^ B lymphocytes was found to be heightened in the glomeruli of nonobese diabetic mice compared to non-diabetic mice, suggesting the potential involvement of B lymphocytes in the pathogenesis and prognosis of DKD. Furthermore, elevated levels of activated dendritic cells were observed in DKD and subsequently validated through flow cytometric analysis (69). Nevertheless, the specific functions of these immune cells, which are increased in DKD, necessitate further investigation.

**Figure 1:**
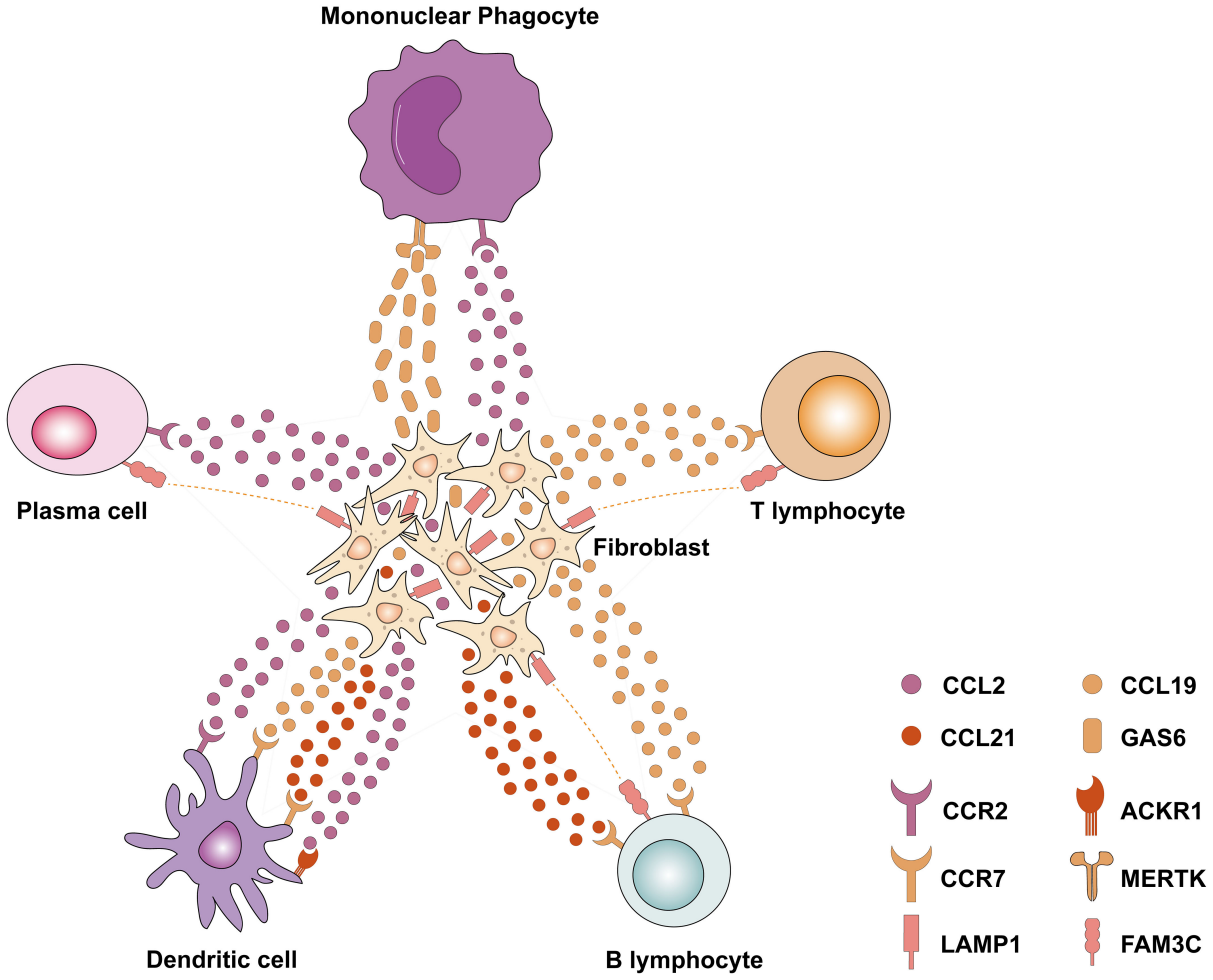
Single-cell transcriptomics revealed the interaction between fibroblasts and various immune cells in DKD. Fibroblasts express chemokines such as CCL2, CCL19, and CCL21, which interact with related receptors (e.g., CCR2, CCR7, and ACKR1) to recruit immune cells to the site of fibrosis. Meanwhile, a variety of immune cells (including plasma cells, B lymphocytes, and T lymphocytes) express FAM3C, which interacts with LAMP1 in fibroblasts and plays an important role in lysosomal biogenesis, autophagy, and cholesterol homeostasis. CCL2, Chemokine (C–C motif) ligand 2; CCL19, Chemokine (C–C motif) ligand 19; CCL21, Chemokine (C–C motif) ligand 21; CCR2, C-C Motif Chemokine Receptor 2; CCR7, C-C Motif Chemokine Receptor 7; ACKR1, Atypical Chemokine Receptor 1; LAMP1, Lysosomal Associated Membrane Protein 1; FAM3C, FAM3 Metabolism Regulating Signaling Molecule C; GAS6, Growth Arrest Specific 6; MERTK, MER Proto-Oncogene, Tyrosine Kinase.

The crosstalk between cells is further elucidated by single-cell transcriptomics, shedding light on the interactions between resident cells and immune cells. The snRNA-seq and snATAC-seq datasets (7) showed that an increase in VCAM1^+^ proximal tubular cells (PT_VCAM1) and infiltrating leukocytes was associated with DKD. PT injury leads to a PT_VCAM1 cellular state that exhibits a pro-inflammatory phenotype characterized by enhanced NF-κB signaling and failed repair. Wu et al. (67) found that diabetes downregulates the spliceosome regulator serine/arginine-rich splicing factor 7 (*Srsf7*) in the PT, and *Srsf7* gene deletion in PT induces a pro-inflammatory phenotype, characterized by highly express genes related to interferon signaling. These studies add evidence in support of the role of the PT in the inflammatory response to DKD at the single-cell level. Lu et al. (71) and Zhang et al. (72) reanalyzed the snRNA dataset by Wilson et al. (69) and demonstrated a substantial increase in potential ligand-receptor pairings between macrophages and endothelial cells and various other cells in the context of DKD. Notably, macrophages demonstrated high interaction scores with neutrophils and epithelial cells in relation to the COL4A3_a1b1 complex, as well as the COL4A4_a2b1 complex. Meanwhile, in a study conducted by Chen et al. (64), differentially expressed genes (DEGs) analysis of glomerular cells, tubular cells, and fibroblasts revealed that DEGs associated with DKD were predominantly enriched in immune- and inflammation-related pathways like TNF, IL-17, and NF-κB pathways. This was further supported by ligand-receptor interaction analyses, which highlighted strong cellular interactions between immune cells and endothelial cells as well as fibroblasts. For instance, T lymphocytes were found to interact with endothelial cells through the CCL5_ACKR1 and PTPRC_MRC1 complexes. Intriguingly, CCL5 and protein tyrosine phosphatase receptor type C (PTPRC) have been identified as crucial immune genes regulating the development and progression of DKD in a separate study (73). Moreover, fibroblasts were found to express elevated levels of chemokines such as CCL2, CCL21, and Lysosomal Associated Membrane Protein 1 (LAMP1), which facilitate interactions with B lymphocytes, T lymphocytes, mononuclear phagocytes, plasma cells, and dendritic cells, ultimately promoting immune cell recruitment and inflammation (**Figure 1**). This crosstalk between immune cells and renal resident cells highlights processes such as upregulation of inflammatory signaling pathways, release of chemokines, and infiltration of immune cells, all contributing to the onset and progression of DKD, particularly in the context of renal fibrosis.

In summary, the predominant involvement of macrophages in DKD is highlighted in current research, with implications of their coexisting pro-inflammatory and anti-inflammatory states within the diabetic milieu. This duality of states may contribute to the underlying progression of the disease. However, the study of other immune cells such as T lymphocytes, B lymphocytes, and dendritic cells has been limited by the constraints of single-cell technology due to their low numbers in the kidney. The existing single-cell transcriptome data of DKD renal tissues, as presented in **Table 3**, has generated a large amount of data. However, further data mining is still pending. There is an expectation that the advancement of algorithms may offer new insights into decoding the mechanisms of immune cells and inflammation in DKD. Moreover, combining advanced technologies such as co-indexed co-detection (CODEX) and spatial histology with single-cell transcriptomics can facilitate a deeper understanding of the roles and mechanisms of immunity and inflammation in DKD.

**Table 3:** Single-cell transcriptomics studies in DKD.

References	Techniques	Model	Tissue type	Samples	Data availability
Fu et al. (2019) (70)	scRNA-seq	STZ-diabetic *eNOS* ^−/−^ mice	Glomerular cells	3 Controls	GSE127235
				3 Diabetic mice	
Wilson et al. (2019) (68)	snRNA-seq	Human DKD samples	kidney cortex	3 Controls	GSE131882
				3 early DKD samples	
Chung et al. (2020) (74)	scRNA-seq	Leptin-deficient BTBR *ob/ob* mice	Glomerular cells	2 control (*ob/+*) mice	GSE146912
				2 *ob/ob* mice (12 weeks)	
				2 *ob/ob* mice (21 weeks)	
Wu et al. (2022) (75)	scRNA-seq	Type 2 diabetic *db/db* mice	Kidney	2 *db/m* mice	GSE181382
				8 vehicle	
				8 SGLT2i	
				6 ARBs	
				6 SGLT2i + ARBs	
Wu et al. (2022) (67)	snRNA-seq	*db/db* mouse with uninephrectomy and renin-induced hypertension mouse model	Kidney	10 *db/m* (control)	GSE184652
				10 *db/db* (vehicle)	
				10 *db/db* + ACEi	
				10 *db/db* + Rosiglitazone	
				10 *db/db* + SGLT2i	
				10 *db/db* + Rosiglitazone + ACEi	
				10 *db/db* +ACEi + SGLT2i	
Wilson et al. (2022) (69)	snRNA-seq	Cryopreserved human diabetic kidney samples	Kidney cortex	6 Controls	GSE131882, GSE195460, GSE151302
				7 DKD samples	
Fu et al. (2022) (7)	scRNA-seq	Type 1 diabetic OVE26 mice	CD45-enriched kidney immune cells	3 Controls (3 months)	GSE195799
				3 OVE26 mice (3 months)	
				3 Controls (7 months)	
				3 OVE26 mice (7 months)	
Collins et al. (2022) (76)	snRNA-seq	*db/db* mice	Kidney	3 Background control	didn’t provide
				5 *db/db*	
Tsai et al. (2023) (63)	scRNA-seq	*db/db* mice	Kidney	3 *db/m* mice (14 weeks)	didn’t provide
				3 *db/db* mice (14 weeks)	
Chen et al. (2023) (64)	scRNA-seq & spatial transcriptomics	Human DKD samples	Kidney specimens	3 Nondiabetic control	Genome Sequence Archive in BIG Data Center (PRJCA015521)
				3 DM	
				3 DKD	
				2 DKD (spatial)	
Liu et al. (2023) (77)	scRNA-seq & Bulk-RNA seq	BTBR *ob/ob* mice	Kidney	8 BTBR WT (6 and 12 weeks)	GSE218563 GSE218086
				8 BTBR *ob/ob* (6 and 12 weeks)	

## Proteomics

Proteomics techniques possess the ability to unravel protein profiles within intricate biological samples, thereby enhancing comprehension of the pathogenic mechanisms underlying DKD. Consequently, this approach emerges as an optimal strategy for identifying a multitude of potential biomarkers associated with DKD.

CKD273, a classic urinary proteome-based classifier originally discovered in 2010, is a panel containing 273 urinary peptides that have been validated in multiple studies (both cross-sectional and longitudinal studies) for early detection of CKD (78–80). Interestingly, the CKD273 contains molecules that are closely related to the pathogenesis of diabetes, including glycoproteins and tubular proteins (81). Meanwhile, the diagnostic performance of CKD273 in DKD has been shown in numerous studies over the years, which may advance the diagnosis of DKD in the near future (81–83). Roscioni et al. (81) analyzed the differential urinary expression of these peptides from the CKD273 classifier, and they found α-2-HS-glycoprotein was closely linked to the worsening of albuminuria in diabetic patients. α-2-HS-glycoprotein is an inflammation-related glycoprotein that is associated with tubular damage in diabetes (81). Another research group revealed that urinary α2-HS-glycoprotein precursor, a calcium-regulatory glycoprotein, was upregulated 2.3-fold in DKD with macroalbuminuria when compared with control subjects (84). The precursor of α2-HS-glycoprotein is considered as a systemic calcification inhibitor, which is associated with inflammation (84). More specifically, inflammation may result in the downregulation of circulating α2-HS-glycoprotein precursor in DKD patients (85). The increased abundance of this glycoprotein in the urine of DKD patients may be associated with more severe inflammatory status.

Fan et al. (86) conducted urinary proteomics and Reactome pathway analysis, and they found that the up-regulated differentially excreted proteins in the urine of DKD patients were enriched in complement cascade, adaptive immune system, and neutrophil degranulation. Using a targeted mass spectrometry method to comprehensively quantify urinary complement protein expression, recent evidence suggested that alteration in urinary complement proteins may serve as a sign of increased inflammatory activity in patients with DKD (87). Zhao et al. (87) conducted targeted and untargeted proteomic analysis of urinary complement proteins in healthy controls (HC), T2DM patients, and patients with biopsy-proven DKD. They discovered that urinary abundance of complement factor H (CFH) was significantly higher in patients with DKD compared with HC participants or patients with T2DM. Further Cox proportional hazards analysis revealed that a higher abundance of urinary CFH was associated with a higher risk of progression to ESRD in patients with DKD. CFH, a 155kDa serum glycoprotein, is mainly synthesized in the liver and acts as the key negative regulator of the alternative complement pathway (88, 89). CFH can not only prevent the formation of the C3 convertase (C3bBb), but also promote the C3bBb dissociation process, leading to the proteolytic inactivation of C3b (90). It was reported that downregulated CFH could lead to altered levels of complement proteins and increased levels of inflammatory mediators including IL-6, IL-8, CCL2, and granulocyte-macrophage colony-stimulating factor (GM-CSF) in an NF-κB dependent way (91). Importantly, recent evidence revealed that CFH plays an important role in protecting the structure and function of renal endothelial cells (92). Dysfunction of CFH potentiates numerous complement-induced renal injuries (93). Targeted deletion of the *Cfh* gene in mice resulted in the activation of alternative complement pathway in the glomeruli, indicating that genetic impairments in *Cfh* are associated with glomerular injury (89, 90). This series of studies provide unprecedented insights into the complement-associated mechanisms underlying the pathophysiology of DKD.

Using the SOMAscan proteomic platform, Kobayashi et al. (94) determined concentrations of 25 TGF-β signaling family proteins in four cohorts with a total of 754 diabetic patients. They discovered that elevated concentrations of neuroblastoma suppressor of tumorigenicity 1 (NBL1), a 165 amino acid secretory protein, in circulation and in urine were highly associated with risk of progression to ESRD. Inflammation is thought to be involved in the progression of DKD to ESRD, but the underlying mechanisms remain largely unknown. Using flow cytometry analysis, Kobayashi et al. discovered that NBL1 was highly expressed in immune cells, such as monocytes, CD4 and CD8 T lymphocytes. Further immunohistochemistry demonstrated that NBL1 was significantly upregulated in proximal tubule epithelial cells in kidney biopsies from patients with DKD. These findings suggest that circulating immune cells under a hyperglycemic state may secrete a large amount of NBL1 into the circulatory system. The secreted NBL1 is then deposited in renal tubules, causing substantial tubular epithelial injury and inflammation, ultimately promoting the progression of DKD. Niewczas et al. (95) conducted proteomics studies aiming at identifying plasma inflammatory proteins associated with the progression of ESRD in diabetic patients. They identified a Kidney Risk Inflammatory Signature (KRIS), comprised of 17 novel inflammatory proteins, significantly associated with the 10-year risk of ESRD. Further prospective study revealed that high concentrations of circulating KRIS proteins potentiate the inflammation underlying ESRD progression in both types of diabetes. Interestingly, the KRIS contains molecules that are mainly involved in innate immune responses, many of which are expressed by monocytes, suggesting that monocytes play a significant role in generating circulating KRIS proteins, and may serve as etiologic drivers of DKD. Additionally, they discovered for the first time that interleukin-15 receptor alpha (IL-15RA) included in the KRIS was involved in inflammation in the context of DKD. IL-15RA was previously considered as a key mediator of several pro-inflammatory signals involved in numerous inflammatory diseases (95, 96). Furthermore, other proteomic studies have also demonstrated that immunity and inflammation are strongly involved in DKD pathogenesis (97, 98). The present data confirmed that proteomics techniques shed light on the important roles of immunity and inflammation in the development and progression of DKD.

## Metabolomics

DM is characterized by heterogeneous metabolic disorders, however, the links between dysregulated metabolism and nephropathy remain elusive (99). Metabolomics is a high-throughput profiling of small molecules (metabolites) that can be the biomarkers for DKD and directly impact disease progression (100). Metabolites derived from cells or gut microbiota are more than just the substrates or products of biochemical processes, but also function as signaling molecules and contribute to inflammation either through effects on pro-inflammatory pathways or via modulation of regulatory proteins (101–103). Thereby, metabolomics, incorporated with microbiomics, transcriptomics, or modification-specific proteomics, provides unprecedented tools to investigate the metabolic regulation of inflammation in DKD.

Over the past decades, metabolomic studies have demonstrated perturbations of metabolic homeostasis associated with or promoting the development of nephropathy in DKD patients. Serum metabolic profiling revealed increased γ-butyrobetaine, symmetric dimethylarginine (SDMA), and decreased azelaic acid in patients, which were significantly correlated to urinary albumin-to-creatinine ratio (104). When these metabolites were applied in the multiple logistic regression model, the area under the curve value for diagnosing DKD reached 0.927. It is reported that elevated SDMA correlates with neutrophilic inflammation by impairing the functionality of endothelial nitric oxide synthase (105). Whereas, the anti-inflammatory property of azelaic acid is well-recognized and serves as the first-line treatment in first-line treatment in acne vulgaris (106). Moreover, urine metabolome found discriminating metabolites included acyl-carnitines, acyl-glycines, and metabolites related to tryptophan (Trp) metabolism that differentiate the progressive and nonprogressive albuminuria in DKD patients (107). Trp metabolism regulates various pathophysiological processes including inflammation, which can be manipulated by gut microbiota, indicating that the dysregulated Trp metabolism in DKD patients may involve dysbiosis (108). Decreased plasma levels of very long-chain ceramide species were observed to be associated with the development of nephropathy in T1DM (109). Multiple studies perceive the very long-chain ceramides as mediators of inflammation and they have protective effects in cardiometabolic disease (110). Another metabolomic research led by Kumar Sharma revealed elevated fumarate levels in the urine of diabetic mice, resulting from the reduced fumarate hydratase (FH) in the diabetic kidneys (both in mice and humans) (111). Recent RNA sequencing and proteomic studies have illustrated that inhibition of FH leads to cytosolic accumulation of fumarate and strong inflammatory effects, by suppressing IL-10 and promoting TNF secretion in macrophages (112). These data suggest a detrimental role of fumarate in DKD via regulating inflammation.

In addition, the microbial metabolite profiling is altered in DKD patients as well, which supports the crosstalk between microbiota and the kidney (113). The microbiota structure is disrupted by the disorder which causes shifts in microbial metabolism (114). The metabolites, including trimethylamine-N-oxide (TMAO), short-chain fatty acids (SCFAs), etc., translocate across the impaired intestinal barrier and fuel metabolic inflammation in DKD (115, 116). The fecal microbiome revealed diminished SCFA-producing strains in patients, which mainly generate butyrate, acetate, and propionate (117). Serum concentration of TMAO increases as nephropathy proceeds (118). TMAO can trigger the activation of inflammatory pathways such as NLRP3, which may be the underlying mechanism of its vicious role in kidney disease (119). SCFA restrains the production of pro-inflammatory cytokines by inhibiting the NF-кB pathway in myeloid cells and promoting the expansion of immunosuppressive regulatory T lymphocytes (120, 121). Treatment with SCFA can combat DKD in diabetic mice by suppressing inflammation and the recruitment of inflammatory cells (122). These studies have provided numerous hints on the potential biomarkers, and the metabolic interactions of host-microbe axes in DKD.

Despite the growing metabolomic studies identifying numerous discriminating metabolites for DKD, few elucidate the pathophysiological mechanism behind these changes. It is unclear whether the altered metabolite levels are the causes or consequences of the amplified inflammation in DKD. Further metabolomic research should be combined with multi-layer omics to address the utility of these metabolites in clinical settings either as therapeutic targets or biomarkers.

## Multi-omics techniques in DKD research: possibilities and limitations

Multi-omics techniques have revolutionized DKD research, which enables us to better understand the pathogenesis of DKD, especially in relation to immunity and inflammation. The application of genomics and epigenetics techniques offers valuable insights into the immune- and inflammation-related genetic factors that contribute to the susceptibility of individuals to DKD (32, 34, 41, 45, 48, 49, 54, 58, 61, 62). Transcriptomics provides a comprehensive perspective on the patterns of gene expression, thereby elucidating the immune- and inflammation-related molecular pathways implicated in DKD (64, 65, 73). Proteomics allows the identification of proteins that are intricately linked with immune responses and inflammation in DKD, shedding light on potential biomarkers and therapeutic targets for DKD (87, 91, 94, 95). Metabolomics facilitates the investigation of small molecules, metabolites, and metabolic pathways associated with the progression of DKD (104, 107, 109, 111, 118–122). The above omics techniques are unbiased and hypothesis-free, allowing for the discovery of novel immune- and inflammation-related factors and pathways involved in DKD, which may not be discernible by using conventional approaches. In addition, the integration of omics data enables network-based analysis, revealing intricate relationships between genes, proteins, and metabolites involved in DKD pathogenesis. However, a major gap hindered the translation of mechanistic insights into clinical breakthroughs. Most omics data failed to be fully interpreted into disease-associated characteristics with experimental validation in animal models or clinical trials. It can be partly attributed to the lack of optimal DKD murine models to recapitulate human nephropathy (26). More importantly, it results from the discrepancy between the “discovery cohorts” that generate the omics data and the “validation cohorts” involved in clinical trials. In most cases, DKD is a clinical diagnosis of DM patients with CKD manifestations in the absence of a diagnostic biopsy (123). Thus, the nephropathy of patients included in DKD cohorts may induced by DM itself or its comorbidities such as hypertension and hyperlipidemia, with distinct pathogenesis (124). Moreover, studies are often compounded by the heterogeneity of disease phenotypes arranged from non-albuminuria to rapid GFR decline (25). These population divergences are also reflected in the different treatment responses to approved drugs in clinical practice, such as SGLT2i (125). Researchers must navigate the challenges associated with data complexity, integration, and experimental validation to maximize the benefits of these powerful omics techniques.

Although current omics techniques offer valuable insights into the pathogenesis of DKD, especially in relation to immunity and inflammation, here are some key questions that remain unanswered in this area: 1) Can omics data be utilized to stratify DKD patients based on their immune-inflammatory profiles to optimize therapeutic strategies and improve patient outcomes? 2) How does the temporal evolution of immune activation unfold in the progression of DKD, and what are the pivotal events that initiate inflammatory responses in diabetic kidneys? We believe that, in future omics studies, integrating multi-omics data with detailed clinical information may help identify patient subgroups with distinct immune and inflammation signatures, guiding personalized treatment strategies for DKD patients. Furthermore, future longitudinal transcriptomic and proteomic studies would help capture dynamic changes in gene and protein expression, helping to delineate the temporal evolution of immune responses in the context of DKD.

## Conclusions and perspectives

In this review, we have discussed the current state-of-the-art omics approaches in DKD research and outlined the tremendous progress in our knowledge of inflammation-related pathogenesis benefiting from these cutting-edge technologies.

However, several significant barriers stand in the way of the clinical translation of omics studies as mentioned above (lack of suitable animal models and inappropriate interpretation of omics data). Some technologies may help to address these challenges. For example, kidney organoids that derive from DKD patients, which more accurately mimic human kidney disease, can serve as powerful tools to dissect omics data (126). Moreover, it also holds the capacity to predict patients’ responses to certain therapies (127). Additionally, advanced algorithms such as machine learning and artificial intelligence could be implemented in these omics studies to make more valid inferences and identify the accessible population (128). More importantly, researchers should carefully discriminate genuine DKD patients from those comorbid with hyperglycemia and other kidney diseases.

**Figure 2:**
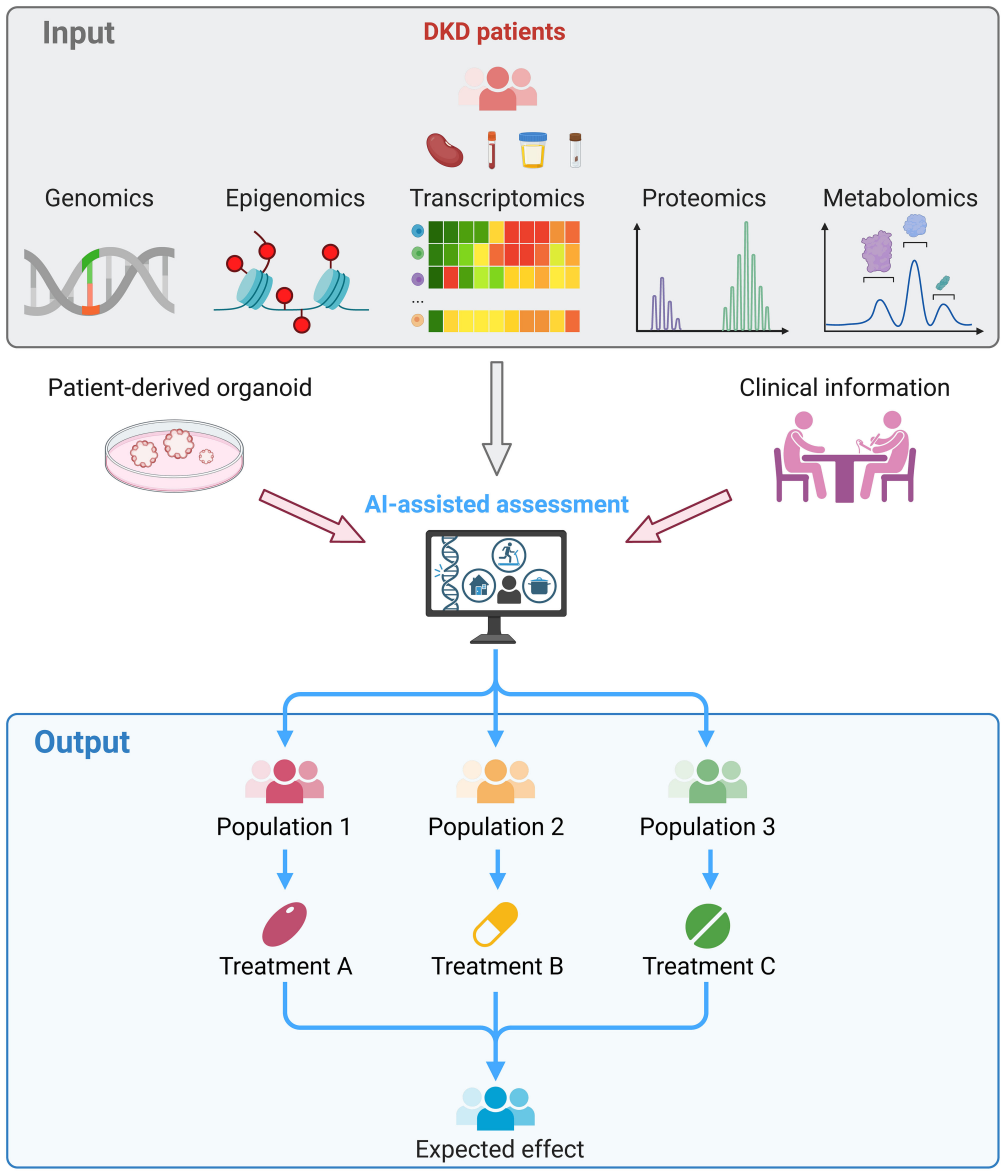
Clinical application of multi-omics in DKD. Kidney, blood, urine or fecal samples were collected from DKD patients and subjected to multi-omics sequencing. The omics data, experimental results of patient-derived kidney organoids, and clinical information of the patient were integrated and assessed by the artificial intelligence, which facilitate to stratify patients. Thereby, treatment that tailored to each patient can be provided and reached the maximum therapeutic effects.

In summary, multi-omics studies improve our understanding of inflammation- and immune-related pathophysiology in DKD with unbiased analysis in a holistic landscape. The further transition of multi-omics approaches from tools for scientific exploration to pipelines for clinical diagnosis and prognosis could lead to the development of precision medicine for DKD. It is expected that the clinical management model for DKD patients will be revolutionized by omics technologies in the future (**Figure 2**).

## Author contributions

XH: Writing – original draft, Writing – review & editing. SC: Writing – original draft, Writing – review & editing. SY: Writing – original draft, Writing – review & editing. WC: Writing – review & editing, Supervision. YZ: Writing – review & editing, Supervision.
